# Delivering integrated child development care in Pakistan: protocol for a clustered randomised trial

**DOI:** 10.3399/bjgpopen17X100677

**Published:** 2017-01-09

**Authors:** Muhammad Amir Khan, Syeda Somyyah Owais, Claire Blacklock, Shirin Anil, Sehrish Ishaq, Shazia Maqbool, Haroon Jehangir Khan, Fareed A Minhas, John Walley

**Affiliations:** 1 Chief Coordinating Professional, Association for Social Development, Islamabad, Pakistan; 2 Project Manager, Association for Social Development, Islamabad, Pakistan; 3 Lecturer in International Public Health, Nuffield Centre for International Health and Development, Leeds Institute of Health Sciences, University of Leeds, Leeds, UK; 4 Project Coordinator and Consultant, Association for Social Development, Islamabad, Pakistan; 5 Project Coordinator, Association for Social Development, Islamabad, Pakistan; 6 Professor of Developmental Paediatrics, Institute of Child Health and The Children’s Hospital, Lahore, Pakistan; 7 Focal Person (Non-Communicable Diseases), Directorate General of Health Services Punjab, Lahore, Pakistan; 8 Head, Institute of Psychiatry, Rawalpindi, Pakistan; 9 Professor of International Public Health, Nuffield Centre for International Health and Development, Leeds Institute of Health Sciences, University of Leeds, Leeds, UK

**Keywords:** primary care, primary health care, general practice, Pakistan, child development, nutrition, depression

## Abstract

**Background:**

Early childhood developmental delay is associated with significant disadvantage in adult life. In Pakistan, high prevalence of developmental delay is associated with poverty, under-nutrition, and maternal depression.

**Aim:**

To assess the effectiveness of an early child development counselling intervention delivered at private GP clinics, in poor urban communities.

**Design & setting:**

A clustered randomised trial in Pakistan.

**Method:**

The intervention was developed following a period of formative research, and in consultation with local experts. A total of 2112 mother–child pairs will be recruited at 32 clinics, from within the locality (cluster); 16 clinics per arm. A primary care counselling intervention (promoting child development, nutrition, and maternal mental health) will be delivered at 6 weeks, 3, 6, and 9 months of the child’s age. Monitoring, assessment, and treatment will also be performed at quarterly visits in intervention clinics. Primary outcome is the developmental delay at 12 months (ASQ-3 scores). Secondary outcomes are stunting rate, and maternal depression (PHQ-9 score). In addition, a process evaluation and costing study will be conducted.

**Discussion:**

This trial will be the first to assess an early child development intervention, delivered in private GP clinics for poor urban communities in Pakistan. If found to be effective, this public–private model may offer a more sustainable, and feasible option for populations in poor urban settings, where private GP clinics are the most accessible provider of primary health care. There is scope for scale-up at provincial level, should the intervention be effective.

**Trial registration:**

The trial has been registered with the Current Controlled Trials ISRCTN48032200.

## How this fits in

In Pakistan, the current mother and child care at private GP clinics does not cover the child brain development and mother mental health components, and nutrition counselling is also suboptimal, mainly due to lack of context-sensitive tools and inadequate staff ability and engagement. The referral linkages with public hospitals are also inadequate for mothers to access an expert consultation for their child development challenges. The intervention offers a set of primary care guidelines and tools for sustainable district health stewardship of private GP clinics to promote optimal early development of urban poor young children (that is, first year of life), through an integrated package of mother and child care. These women currently fail to access early child development care because of overloaded public hospitals, the inability of unregulated private clinics, and lack of affordability. This ‘action’ will solve the problem by making early child development care package available at selected private GP clinics. The care package will have three main components: nutrition, child development, and maternal mental health.

## Introduction

Childhood developmental delay is associated with poor subsequent educational attainment, and lower income during adult life, contributing to a cycle of poverty. It is a significant public health concern in many low- and middle-income countries, including Pakistan.^1^

Prevalence of developmental delay in Pakistan has been estimated at around 15%,^2^ even exceeding 30% among children from poorer families.^3^ Developmental delay is strongly linked to undernutrition,^4–6^ and maternal depression.^7^ Indeed, inadequate stimulation in early childhood, and maternal emotional characteristics, are associated with poorer cognitive development.^4^ In one Pakistani study more than one-quarter of new mothers had depression.^8^

There is evidence from low resource settings that programmes to improve infant stimulation and enhanced parenting skills have a beneficial effect on development.^9–11^ In the Caribbean, a parenting intervention integrated into routine primary care was effective in improving cognitive development.^12^ In Pakistan, a development and nutrition intervention delivered by community ‘lady health workers’ in Sindh province, had a significant positive effect on development outcomes.^13^

In poor urban areas of Pakistan, public primary care services are scant, and referral hospitals difficult to access due to distance, expense, and high demand. Primary care is therefore commonly provided by private GP clinics and small community hospitals. These are unregulated, unsupported, and vary in both quality and cost of services. Existing maternal and child health services do not adequately meet the early child development and maternal mental health needs of the population, and nutritional counselling is lacking.^14^ This trial has been designed to investigate the effectiveness and feasibility of integrating an early child development intervention into existing private GP clinic services for poor urban communities, and the aim will be to assess the effectiveness of a primary care intervention in reducing early developmental delay, in poor urban communities of Pakistan, using public–private partnership.

## Method

### Study design

**Figure 1.**
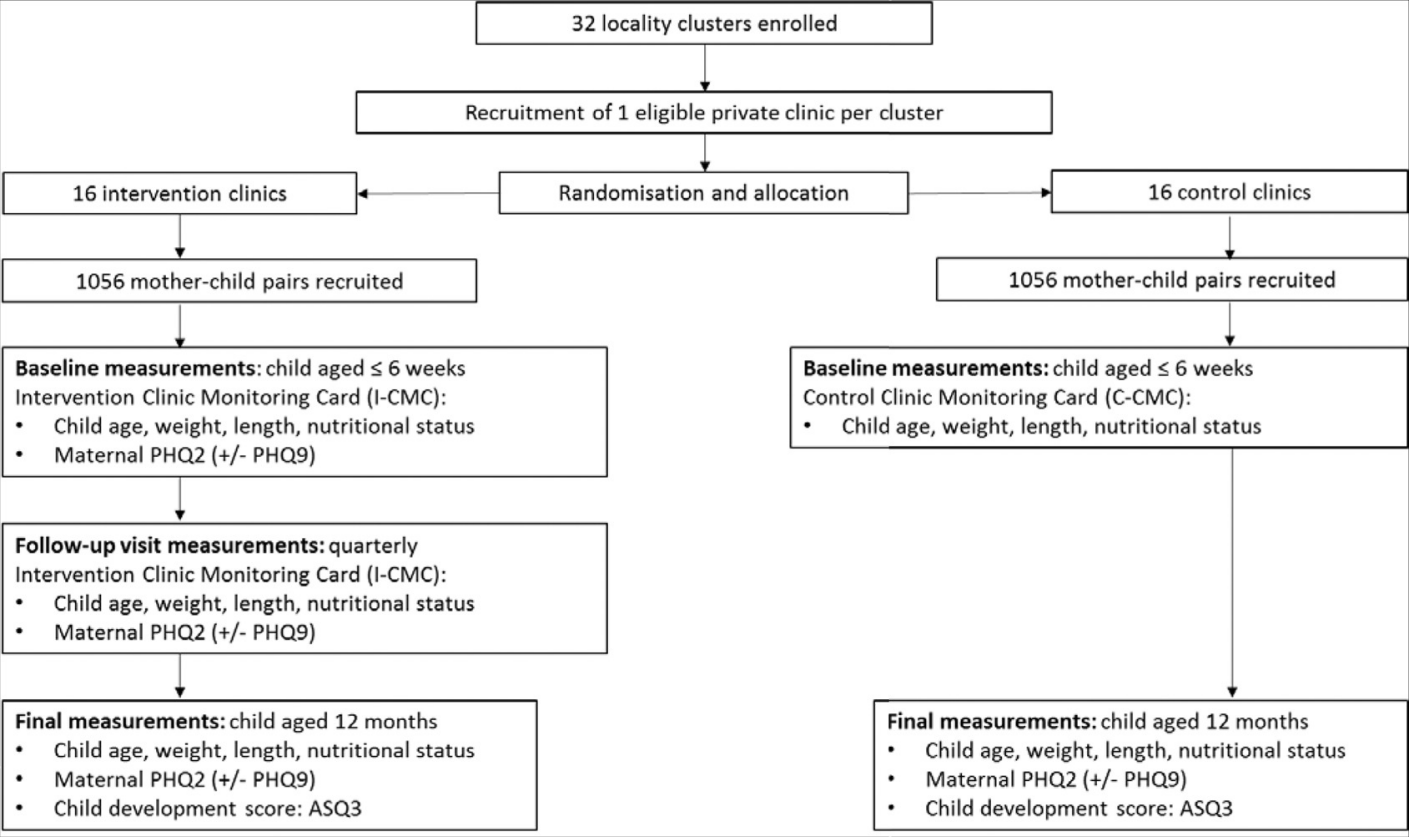
Trial flow diagram

Pragmatic, parallel arm, clustered randomised controlled trial with two arms (intervention and control) will be conducted to compare an early child development intervention delivered in private urban GP clinics in Pakistan, with usual routine care. Thirty-two clusters will be randomised using a 1:1 ratio, to intervention or control Figure 1.

### Setting

The districts of Lahore and Rawalpindi are study sites, following consultation with the Directorate General Health Services (DGHS), Children’s Hospital, and the Institute of Psychiatry. Lahore and Rawalpindi have large urban poor communities, and an extensive existing network of private GP clinics and small private hospitals. Average household size is 7.2 and 6.5 in Lahore and Rawalpindi respectively. Female literacy is around 60% in both districts. Electricity is available to more than 90% of households, but piped water to only 75% and 41% in Lahore and Rawalpindi respectively (and gas for cooking, 63% and 53%).^15,16^

### Clusters

Each trial cluster will represent one priority poor urban locality settlement, with limited access to a public hospital, and an average population of 50 000.

### Trial clinics

Within each cluster, one study clinic will be selected from available private GP providers, with preference for longer established clinics, and existing providers of maternal and child care. Private clinics typically comprise one doctor (GP), and employ at least one clinic assistant. Clinic assistants are usually males from the local area, with between 10–12 years of schooling, but no formal paramedic training.^14^ They are trained on-the-job by the private doctor, and support the private doctor by dispensing medication and performing other tasks, as necessary.

### Recruitment of mother–child pairs

Eligible mother child pairs will be recruited at participating private clinics by clinic assistants trained in trial recruitment and consent procedures. Recruitment will continue up to 6 weeks of child’s age, to allow for cultural restrictions during first 40 days postpartum.

#### Inclusion criteria

- Mother–child pair
- Child ≤6 weeks

#### Exclusion criteria

- Prematurity (<36 weeks gestation), history of congenital abnormality, delayed cry at birth, seizures, cretinism, or low birth weight (<2500 g)
- Mother intends to move out of area during study period
- Child not accompanied by mother (for example, accompanied by another relative)

### Intervention

The intervention was developed following a period of formative research.^14^ In brief, this focused on understanding the context within private GP clinics, and experiences and preferences of mothers. Feasibility and acceptability of proposed intervention components were explored, and findings used to inform intervention development. The outline is described in Box 1.

#### Maternal counselling (core intervention)

A quarterly maternal educational counselling session with the clinic assistant at the private GP clinic will promote early child development, nutrition, and maternal mental health. Counselling content is developed from existing guides, in consultation with technical experts,^17,18^ supported by a pictorial flipbook.

Counselling will encourage mothers to engage in activities, appropriate for the expected stage of child development. It will also focus on nutrition and maternal mental health messages. Content was adapted for the context in Pakistan through a process of expert consultation. Trained and experienced artists were engaged to illustrate key activities and messages. The pictorial flipbook is two-sided: one side with pictures of activities to be shown to the mother; and the other with prompts for the clinic assistant. Each counselling session comprises 4–5 pages of the flipbook, and takes no more than 10 minutes to deliver.

Mother–child pairs will be invited to return for follow-up every 3 months, to receive the next counselling session; that is, at 6 weeks, then at 3, 6, and 9 months of the child’s life.

#### Assessment and treatment of child and mother

At each visit, the clinic assistant will monitor the growth of the child, and screen for maternal depression (PHQ-2 score).^19,20^ Referral will be made to the private GP for further assessment where indicated.

Where indicated, the private GP will assess the child for malnutrition and developmental delay, and the mother for depression using the PHQ-9 score,^19,20^and manage any concerns, including referral to a specialist if necessary.

#### Ancillary intervention components

**Box 1.** Early child development care in private GP clinics

	Control clinics	Intervention clinics
**Mother–child care**	Usual care	Standard counselling session on childhood nutrition, development, and maternal mental health, using pictorial flipbook. Monitoring and screening of child growth and maternal mental health. Assessment and treatment (including referral to specialist) of childhood nutrition, development, or maternal depression. Follow-up of mother–child pairs in clinic at 3, 6 and 9 months (including SMS or telephone reminder, if required)
**Low dose vitamin A**	Yes	Yes
**Community advocates**	Yes	Yes
**Branding of clinic by Directorate General Health Services**	Yes	Yes
**Training of private doctors (GPs) and clinic assistants**	Control clinic staff will receive basic training only, focused on the correct use of study recording forms, with a general overview of the importance of childhood development and maternal health; that is, they will not receive any specific training on intervention activities.	Clinic assistants will be trained by project field coordinator (under supervision of project manager and specialist doctor) using the study clinical assistant training protocol (CATP), which includes: how to conduct a standardised counselling session using the flipbook; how to administer the PHQ-2; and how to measure and record child length and weight. Private GPs will be trained by an experienced specialist according to the study private doctor training protocol (PDTP), which includes: clinical management of children with malnutrition and developmental delay in the private clinic setting; how to use PHQ-9 for the diagnosis of maternal depression; and how to assess the mother–child pair for a specialist referral, when required, to the appropriate public tertiary care facility. The training protocols, both CATP and PDTP, are developed by a group of local experts and specialists, adapted from international best-practice guidelines and standards.^16,17^ Training will last approximately 2 hours, and will include a mixture of explanation by the project field coordinator, and role-play exercises by participants.

Maternal counselling, and assessment and treatment, will be supported by:

- Training of private GPs and clinic assistants
- SMS reminders to mothers for follow-up visits

A logic model and theory of change for the intervention is described in Box 2, drawing on the refined Theoretical Domains Framework.^21^

**Box 2.** Logic model for proposed intervention mechanisms/theory of change (adapted from Bonell and colleagues 2015)^38^

	**Inputs**	**Processes and actions**	**Intended changes (Theoretical domains framework)^21^**	**Intended outputs**	**Intended health outcomes**
**Intervention**	Formative research: interviews with providers, mothers, and key stakeholders Consultation with experts to develop technical materials: • Training protocols and manuals • Counselling flipbook • Community advocate leaflets Training of private GPs and clinic assistants: theoretical and practical training	Quarterly counselling sessions for mothers (child development, child nutrition, maternal mental health) Quarterly child growth monitoring Text message/SMS reminders to mothers for quarterly follow-up appointments Assessment and treatment of chid developmental delay (may include referral) Assessment and treatment of maternal depression (may include)	**Mother** Social influences Knowledge Beliefs about consequences Environmental context and resources Reinforcement **Private GP and clinic assistant** Social influences Knowledge Skills Environmental context and resources Social and professional role and identity	**Mother** Mother–child health services are accessible and desirable to mother Increased capability and confidence to support child nutrition and development in the home environment Increased capability and confidence to protect own mental health in the home environment **Private GP and clinic assistant** Motivated and skilled to provide mother–child health services	Reduced developmental delay at 12 months of age (primary outcome) Improved childhood nutrition Reduced maternal depression
**Both intervention and control arms**	DGHS endorsement of intervention and control clinics Identification and orientation of community advocates Growth Monitoring Cards to intervention and control clinics Standard calibrated equipment to intervention and control clinics Supervision of data entry on Growth Monitoring Card by clinic assistant (Field research officer)	Recruitment of mothers by community advocates Vitamin A supplement		Adequate participant recruitment High quality data	

### Public–private partnership

Both intervention and control clinics will be endorsed by the DGHS to advertise the availability of early childhood development care to the community.

### Community advocates

Participation of mother–child pairs in the study will be encouraged by institution-based members of the community (such as the barber shop or grocery store) advocating for clinics, including distributing study leaflets to interested clients.

### Control clinics

Control clinics will continue usual mother–child care. Control clinic staff will receive basic training only, focused on the correct use of study reporting forms, and a general overview of childhood development and maternal health, that is no training in counselling, or in assessment and treatment of mother–child pairs, or provision of flipbook.

Both intervention and control clinics will receive standardised calibrated equipment (digital scales, and infantometer), to ensure quality of study data. Clinic assistants will be instructed to maintain records in kilograms and centimeters, to ensure uniformity in measurements. Intervention and control clinics will receive a small monthly reimbursement, for participation in research activities.

### Outcomes

#### Primary outcome

##### Suspected developmental delay on screening: ASQ-3 score at 12 months

The Ages and Stages Questionnaire 3^rd^ Edition (ASQ-3) is a childhood developmental screening tool.^22^ By assessing across five developmental domains, it identifies children in primary care who are eligible for further assessment. It is intended that the parent completes the questionnaire, after observing the child at home. However, it can be adapted, for example a parent can be assisted if literacy is poor. Translation and cultural adaptation of the questionnaire is also recommended.^23–25^ Internal and external validity trials of ASQ in a range of populations, cultures, and settings report mixed findings.^24,26–30^ The ASQ-3 questionnaire for children aged 12 months is reported to have a sensitivity of 90% and specificity of 87.5%, when compared to equivalent cutoffs using the Battelle Developmental Inventory-II.^31^ A trial of Hindi ASQ-3 questionnaires in a South Asian population of high and low risk children, found an overall sensitivity of 83.3% and specificity of 75.4%.^32^ The ‘home procedure’ was feasible in India when delivered by non-psychologists, under instruction and supervision. Inter-observer reliability was good. Overall internal validity of the questionnaires was also good, but varied at subscale level.^25^

The ASQ-3 questionnaire has been translated into the Urdu language and adapted to local context for this study. ASQ-3 assessors will have a bachelor’s degree in psychology, and receive training and supervision. Assessment will be conducted at 12 months of age, in private GP clinics. The assessor will record: 1) mother’s responses to the ASQ-3 questionnaire, based on recall and any self-initiated observations; and 2) the assessor’s own on-site observations of specific activities during the assessment session, making use of appropriate toys and props. Mother and assessor scores will be analysed separately, and inter-rater agreement assessed. Final ASQ-3 outcome score will be a composite score of mother response and on-site observations.

#### Secondary outcomes

##### Childhood nutrition status: weight (kg, percentile), length (cm, percentile) at 12 months

Each child will be measured for weight and length by the clinic assistant. Measurements will be plotted on a growth chart, and percentiles calculated. The prevalence of underweight and stunting will be calculated.

##### Maternal depression: PHQ-9 score at 12 months

Maternal depression will be assessed at 12 months by administering the PHQ-9 questionnaire, already available in the Urdu language.^33^ PHQ-2 and PHQ-9 are validated screening tools for depression.^19,20^

#### Data management

##### Participant registration and monitoring data

Sociodemographic information will be recorded at participant enrollment. Ongoing clinical data will be collected by the clinic assistant. Completeness of data collection will be checked by a research officer, who will update trial records, kept securely at the research office. Anonymised electronic data will be entered into SPSS Version 20.0, stored on a password protected computer.

##### 12-month outcome data

All mother–child pairs will be invited to a 12-month final outcome assessment visit. Hard copies of outcome assessment forms will be kept securely, and anonymised electronic data inputted into SPSS (version 20.0) for analysis.

#### Analysis

Both individual and cluster level analysis will be conducted according to intention to treat in SPSS version 20.0 and R version 3.2.3 respectively. Intervention and control arms will be compared at baseline to ensure equal distribution of potential confounders like maternal education, the purpose of randomisation. Scores will be calculated for the five domains of developmental delay namely gross motor, fine motor, problem solving, and personal–social using ASQ3. The two arms will be compared for these five domains of development delay at 12 months by independent sample *t*-test adjusting for clustered data, as used by other researchers,^34^ by applying t.test.cluster in Hmisc package in R. Stunting will be measured at end line using WHO Anthro SPSS Macros. The two arms will be compared with respect to stunting by χ^2^ test adjusted for clustering^35^ by applying donner function in aod package in R.^36^ Mother’s mental health condition will be compared across both arms as end line using PHQ-9 scores *t*-test adjusting for clustered data.^34^ A *P*-value <0.05 will be considered significant. Generalised estimating equation (GEE) and generalised linear mixed models (GLMM) will be applied using R packages gee and glmmML respectively in case of residual confounding.^37,38^

#### Sample size calculation

At least 1056 mother–child pairs will be required (in 16 clusters: 66 mother child pairs per cluster) in each of the intervention and control arms (a total of 2112 mother–child pairs in a total of 32 clusters) to detect a difference of 20% in development delay (assuming 33% suspected development delay^39^ at baseline) at 80% power, 5% level of significance and an intra-class correlation coefficient of 0.15 for maternal education,^13^ allowing for 10% loss to follow-up in each arm.

#### Randomisation

Simple randomisation will be performed by drawing from a hat, by a member of the host research organisation not involved in the study. Stratification by districts (Rawalpindi and Lahore) has not been done as both share similar characteristics being major cities in the province of Punjab. Nevertheless clinic size, if not equally distributed between both arms by randomisation, will be taken care of in modeling (GEE and GLMM as mentioned above).

#### Blinding

Due to the nature of the intervention, participants will not be blinded. ASQ-3 assessors will be blinded to allocation of participants. Members of the research team undertaking 12-month maternal PHQ-9 assessments will not be blinded.

#### Ethics and dissemination

Verbal consent will be obtained from community leaders. Written consent will be taken from the clinic staff, and verbal consent from participating mothers. Mothers can withdraw consent at any time. Clinical referrals will be made to local specialist services where appropriate.

The trial results will be published in open access peer-reviewed journals. Results will be communicated to key government and non-government stakeholders at national level, with a view to scale-up if the intervention is effective, feasible and acceptable.

The SPIRIT checklist has informed the preparation of this protocol.^40^

#### Process evaluation

A process evaluation will accompany the trial, following MRC Guidance.^41^ Mixed methods will be used to explore intervention implementation, mechanisms of impact, and context. Quantitative data will be extracted from study records, and descriptive analyses presented. Qualitative data will be gathered from in-depth interviews. Purposive sampling will be used, for maximal variation in views and experience. Interviews will be audiorecorded and transcribed in Urdu. Framework Approach will be used in analysis.^42^

#### Economic evaluation

Data will be collected on costs incurred by the intervention to inform incremental cost-effectiveness ratio (ICER) analysis of the health services cost. The study will account for both provider and client perspectives, and will consider recurrent costs incurred should the intervention be implemented without study resources. The marginal costs of the intervention, to both public and private providers will be estimated, as well as costs to participating mothers. An economic survey of participating mothers will be conducted at 12 months to determine costs of care.

## Discussion

### Summary

A clustered randomised controlled trial will assess the effectiveness of an early child development counselling intervention delivered at private GP clinics, in poor urban communities of Pakistan. Primary outcome is the developmental delay at 12 months (ASQ-3 scores). Secondary outcomes are stunting rate, and maternal depression (PHQ-9 score).

### Strengths and limitations

The intervention was developed in consultation with local experts, following a period of formative research. It will be implemented in the existing private GP clinic setting, in the context of a public–private partnership. Anticipated challenges of the proposed research include:

- Follow-up appointments are not usual practice in the existing primary care context in Pakistan. Non-adoption by providers and patients is a therefore a possible challenge. Some trial supervision will be provided to clinics by research staff, however the aim is to assess real-life implementation. Non-attendance by mothers for follow-up visits may lessen measured effectiveness of the intervention.
- Most patient records are stored in primary care for a maximum of 3 days, if at all. Both intervention and control clusters will be instructed to keep clinic records, which is a new concept, and may present challenges.
- Providers may not implement the intervention according to the trial protocol, for example if there is insufficient time during the clinic visit. This may reduce the effectiveness of the intervention, and will be explored in the process evaluation.
- Mothers in the intervention arm will be more familiar with child development activities than those in the control arm, which may bias recall of developmental achievements at 12 months. On-site observations of development will to try to reduce this bias.

### Comparison with existing literature

To the authors’ knowledge this study is the first to assess an early child development intervention, delivered in private GP clinics in Pakistan.

### Implications for practice

Private sector engagement in education and healthcare delivery is a national strategy for universal coverage of health and social services in Pakistan, and the proposed intervention offers an alternative to a home-based delivery model of early child development care. If effective, service provision at private clinics may offer a more feasible option for populations in poor urban settings, where they are by far the most accessible provider of health care.

## Trial registration

The trial has been registered with the Current Controlled Trials ISRCTN48032200.
